# Durable response of therapy-related MDS/AML with concomitant Waldenström’s macroglobulinemia treated with venetoclax and azacitidine

**DOI:** 10.1007/s00277-022-04777-z

**Published:** 2022-02-21

**Authors:** Mitsuhito Hirano, Mikako Tamba, Norihito Inoue, Michiru Ikegami, Naoki Onda, Yuko Shirouchi, Yuko Ishihara, Hitoshi Abe, Noriko Nishimura, Yuko Mishima, Kengo Takeuchi, Dai Maruyama

**Affiliations:** 1 Department of Hematology Oncology, The Cancer Institute Hospital of Japanese Foundation for Cancer Research, 3-8-31, Ariake, Koto-ku, Tokyo 135-8550 Japan; 2 Division of Pathology, The Cancer Institute, Japanese Foundation for Cancer Research, 3-8-31, Ariake, Koto-ku, Tokyo 135-8550 Japan; 3 Department of Clinical Laboratory Medicine, The Cancer Institute, Japanese Foundation for Cancer Research, 3-8-31, Ariake, Koto-ku, Tokyo 135-8550 Japan; 4 Pathology Project for Molecular Targets, The Cancer Institute, Japanese Foundation for Cancer Research, 3-8-31, Ariake, Koto-ku, Tokyo 135-8550 Japan; 5 Department of Pathology, The Cancer Institute Hospital, Japanese Foundation for Cancer Research, 3-8-31, Ariake, Koto-ku, Tokyo 135-8550 Japan

Dear Editor,

Venetoclax is an oral drug that is BH3-mimetic and a selective B-cell lymphoma 2 (BCL-2) inhibitor. Inhibition of BCL-2 induces apoptosis in hematological malignancies [1]. Venetoclax has shown activity in patients with acute myeloid leukemia (AML) [2, 3] and chronic lymphocytic leukemia [4]. The approval of venetoclax is suggested to expand the treatment options for hematological malignancies. Herein, we report a patient with therapy-related myelodysplastic syndrome/acute myeloid leukemia (t-MDS/AML) with concomitant Waldenström’s macroglobulinemia (WM) who was treated with venetoclax and azacitidine (Ven/Aza).

**Fig. 1:**
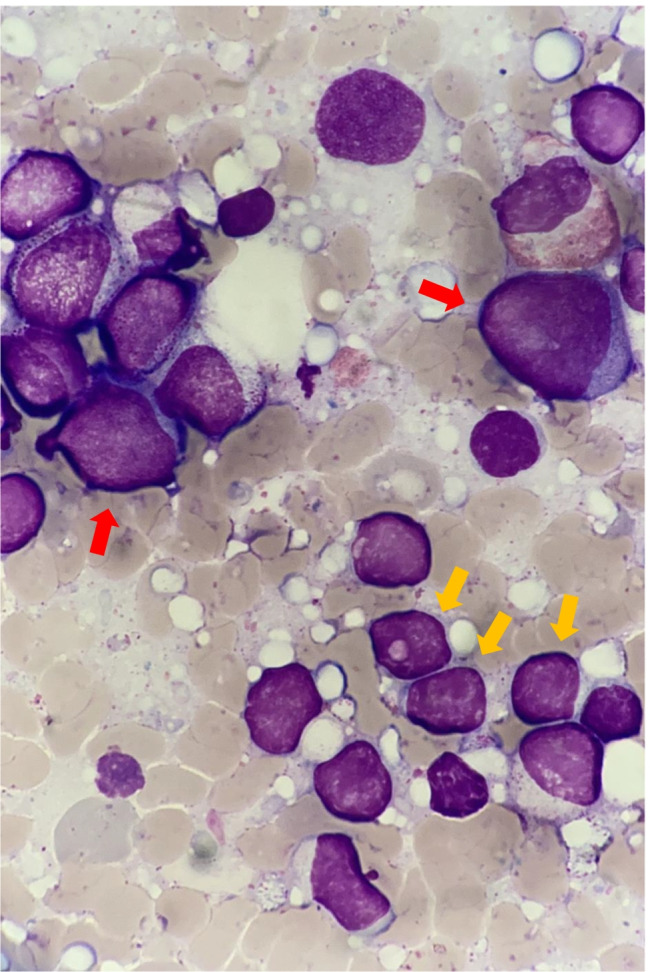
Bone marrow findings in the patient with t-MDS/AML and WM. Lymphoplasmacytoid cells, which have a slightly eccentric nucleus (yellow), and blast cells (red) were found in the patient’s bone marrow

A 74-year-old male diagnosed as having WM more than 20 years previously had been administered several cytotoxic agents including MP (melphalan, prednisolone, every 3 weeks) at a previous hospital, R-ICE (rituximab 375 mg/m^2^, day 1, ifosfamide 1.7 g/m^2^, days 2–3, 1.6 g/m^2^, day 4, carboplatin 5 × AUC, day 2, and etoposide 100 mg/m^2^, days 2–4, for 3 cycles), and rituximab (375 mg/m^2^, 13 times for 2 years). The IgM level decreased from 3270 to 2020 mg/dL after these treatments. We assessed him as a minor response according to the International Workshops for WM (IWWM) criteria [5]. After that, he was followed by observation via watch-and-wait for about 2.5 years. As his WM progressed, he received tirabrutinib, a novel Bruton’s tyrosine kinase (BTK) inhibitor that has been approved in Japan for WM [6]. For the first month, he was treated with tirabrutinib 600 mg once daily, but due to developing of pneumonia, dosage was reduced to 160 mg once daily. After administration of tirabrutinib, the symptoms of WM, such as fever and fatigue, improved and serum IgM levels decreased. The level of IgM in the pre-treatment was 4018 mg/dL; after treatment, the IgM level decreased to 548 mg/dL. The response was assessed as partial response according to the IWWM criteria [5]. However, after 5.5 years of starting tirabrutinib, blast cells appeared in the peripheral blood (PB) and bone marrow examination revealed that t-MDS arose (blast 14.4%) and there was infiltration of lymphoplasmacytoid cells (Fig. 1). Karyotypic G-banding showed complex karyotype abnormalities such as 46,XY,del(20)(q11.2q13)[13/20]/48,idem,add(1)(p34),del(2)(q?),-5,del(5),del(5)(q?), + add(7)(q11.2), + 8,-11,-15, + 19, + r1, + mar1[7/20]. None of the specific chimeric genes was found. One week later, blast cells in PB exceeded 20% and his t-MDS developed overt t-AML. The complete blood count in PB, white blood cells (WBC), neutrophil (Neu), hemoglobin (Hgb), and platelet (Plt) were 10,300/μL, 1030/μL, 10.7 g/dL, and 79,000/μL, respectively. He was AML with myelodysplasia-related changes as WHO classification, and AML M2 as FAB classification. The patient has adverse risk according to European LeukemiaNet stratification for the reason that he had complex karyotype. Tirabrutinib was discontinued and CA (cytarabine 10 mg/m^2^, days 1–14, and aclarubicin 14 mg/m^2^, days 1–4, for 1 cycle) was administered for t-AML. Although complete remission with incomplete hematologic recovery (CRi) was achieved, fever, serum IgM elevation, and splenomegaly progressed. WBC, Neu, Hgb, and Plt were 3200/μL, 300/μL, 7.6 g/dL, and 49,000/μL. The IgM level rose to 1791 mg/dL. It was thought that cytarabine and aclarubicin controlled t-MDS/AML but not WM. Rituximab (375 mg/m^2^, weekly) was administered as treatment of WM, but no response was observed. He developed bilateral cataplexy of brachial and antebrachial arms and was suspected to be Bing-Neel syndrome due to WM progression [7]. The baseline, WBC, Neu, Hgb, and Plt were 4100/μL, 860/μL, 6.6 g/dL, and 45,000/μL, respectively. He developed anemia and needed red blood cell transfusions for two or three times in a week. We administered tirabrutinib retreatment, and fever, splenomegaly, and neurologic symptoms improved; however, anemia progressed and blast cells reappeared in PB. WBC, Neu, Hgb, and Plt were 2100/μL, 230/μL, 5.7 g/dL, and 41,000/μL, respectively.

At this time, because Ven/Aza [2] was approved as a treatment for AML in Japan, he was treated with Ven/Aza (venetoclax 400 mg/body, days 1–28 and azacitidine 75 mg/m^2^, days 1–7). Hyperuricemia and hyperphosphatemia associated with laboratory tumor lysis syndrome were found, but these abnormalities improved. After initiation of Ven/Aza treatment, blast cells disappeared and anemia was improved. In addition, splenomegaly tended to improve and serum IgM levels decreased. CRi was achieved for t-AML and PR was achieved for WM. WBC, Neu, Hgb, and Plt were 2100/μL, 290/μL, 7.0 g/dL, and 70,000/μL, respectively. Treatment of both t-MDS/AML and WM by Ven/Aza is ongoing.

Venetoclax has been suggested to be effective in the treatment of AML, including t-AML [2], and B-cell malignancies including WM [8, 9]. A phase II trial (NCT02677324) evaluating venetoclax in 30 WM patients of whom 15 were previously treated by BTK inhibitors showed overall response rate was 90%, and PFS rate was 82% with a follow-up of 18 months [8]. In these reports, venetoclax monotherapy once daily (800 mg) was administered [8, 9]. In our patient, Ven/Aza according to the VIALE-A study [2], a combination of venetoclax and azacitidine, was also effective against refractory WM.

In conclusion, further data is needed; however, our study suggests that Ven/Aza is an effective treatment regimen for patients with t-MDS/AML with concomitant WM.
